# Increased mRNA expression of key cytokines among suspected cases of *Pneumocystis jirovecii* infection

**DOI:** 10.1186/s12879-020-05729-6

**Published:** 2021-01-07

**Authors:** Mohammad Y. Alshahrani, Mohammed Alfaifi, Mesfer Al Shahrani, Abdulaziz S. Alshahrani, Ali G. Alkhathami, Ayed A. Dera, Irfan Ahmad, Shadma Wahab, Mirza M. A. Beg, Ali Hakamy, Mohamed E. Hamid

**Affiliations:** 1 Department of Clinical Laboratory Sciences, College of Applied Medical Sciences, King Khalid University, Abha, Saudi Arabia; 2 Department of Medicine, Faculty of Medicine, Najran University, Najran, Saudi Arabia; 3 Department of Pharmacognosy, College of Pharmacy, King Khalid University, Abha, Saudi Arabia; 4 Department of Biochemistry, Maulana Azad Medical College, New Delhi, India; 5 Respiratory Therapy Department, Faculty of Applied Medical Sciences, Jazan University, Jazan, Saudi Arabia; 6 Department of Microbiology and Clinical Parasitology, College of Medicine, King Khalid University, Abha, Saudi Arabia

**Keywords:** Immune-compromised, Interleukins (ILs), Immune-fluorescent staining, PCR, Saudi Arabia

## Abstract

**Background:**

Pneumocystis pneumonia (PCP) is a fatal infectious disease caused by *Pneumocystis jirovecii* (PJP). The major factor relevant to morbidity and mortality seems to be the host inflammatory reaction. The objective of this study was to evaluate the role of IL-2, IL-4, IL-10, and IL-13 cytokine mRNA expression among suspected *P. jirovecii* infection.

**Methods:**

This was a cross-sectional analytical study undertaken in Aseer region, Saudi Arabia. One hundred suspected PCP cases and 100 healthy controls were included in the study. Basic clinical manifestations, radiological findings, microbiological and immunological findings were extracted from the hospital records from January 2019 to August 2019, Pneumocystis detection was done by immune-fluorescent staining (IFAT, Gomorimethanamine silver staining (GMSS), Giemsa staining, Toluidine blue O (TBO), and Pneumocystis RT-PCR.

**Results:**

Increased more than 5 fold, 3 fold, 4 fold, and 7 fold of IL-2, IL-4, IL-10, and IL-13 mRNA expression were observed in PCP cases compared to controls. Higher expression of IL-2 mRNA was connected with crept, wheezing and chest X-ray findings like central perihilar infiltrate, patchy infiltrate, consolidation, hilar lymphadenopathy, pneumothorax, pleural effusion which showed higher expression compared to counterpart (*p*< 0.0001). Higher expression of IL-4 mRNA was found to be significantly associated with weight loss (*p*=0.002), dyspnea (*p*=0.003), crept (*p*=0.01), and chest X-ray findings (*p*< 0.0001). Significantly increased expression of IL-10 mRNA was observed to be associated with weight loss, dyspnea, night sweats, wheezing, and different findings of chest X-ray compared to their counterparts, whereas, IL-13 mRNA was observed in cases with fever. Suspected cases of PCP confirmed positive by IFTA with higher IL-2, IL-4, and IL-10 mRNA expression compared to negative cases. RT-PCR confirmed PCP cases had significantly higher expression of IL-2, IL-4, and IL-10 as well as IL-13 mRNA compared to negative cases. Positive detected cases by GMSS showed higher IL-2, IL-10 mRNA expression, while Giemsa showed only higher IL-4 mRNA expression compared to negative cases.

**Conclusion:**

Confirmed cases of *P. jirovecii* showed higher IL-2, IL-4, IL-10, and IL-13 mRNA expression comparatively to negative cases. Increased expression of cytokines may be indicative of infection severity and could help in patients’ management.

## Background

Pulmonary infection, exclusively PCP, is a substantial contributing agent for disastrous diseases [1]. *P. jirovecii* is the contributing cause of PCP [2]. PCP has been a potentially lethal disease in immune-compromised patients [3]. The clinical appearance of PCP cases comprising clinical and radiological variability, management response, and consequences can be diverse broadly [4]. Several appearances of evidence recommend that the foremost causative factor for morbidity and mortality of PCP patients seems to be the host inflammatory reaction to infectious organisms rather than the organism itself [5].

Type-1 immune response encompasses the generation of Th1 cells-related cytokines, arousing macrophage beginning, the genesis of cytotoxic CD4+ T cells, and the release of antibodies for opsonizing, delayed-type hypersensitivity [6, 7].

Th1 is a group of indispensable cells that participated in cytokine production, cell-mediated inflammation, delayed-type hypersensitivity responses, and reflection to be crucial for immunity against intracellular pathogens [8–10]. IL-2 stimulates activation of macrophage cytotoxic CD4+ T cell generation, antibody production for opsonizing, and T cell-mediated hypersensitivity reactions [11]. The generation of IL-10 is, therefore, vital for the control of Th1-mediated immune pathology as well as molecular proceedings that control its stimulation and numerous cytokines increase IL-10 levels by Th1 cells [12], and Interleukin-4 (IL-4) has also been involved in causing the up-regulation of IL-10 production [13]. IL-10 participates in the regulation and expansion of Th cells and innate immune responses [6–8].

Th2 cells participate in the defense system against multicellular parasites and their contribution in allergies and atopic illnesses, Th2 cells function has been observed in epithelial tissues, especially the intestinal tract and lungs [14]. Th2 cell population is best documented for the production of IL-4, IL-5, and IL-13 [15]. IL-4 is a multifunctional, pleiotropic cytokine, which is primarily secreted by activated Th2 cells, as well as by mast cells, basophils, eosinophils, and γδT cells [16]. In the adaptive immune system, IL-4 has been a critical endurance factor for lymphocytes and B cells; it encourages plasma cell differentiation and IgG1 and IgE antibody switching [17]. Th1 and Th2 cells contribute to adaptive immunity as well as the role of regulatory T cells (Treg), which has been established against fungal infection. Treg cells diminish Th1 response, reduce inflammation, promote infection tolerance, and induce reinfection resistance [11]. The phagocytic antifungal action involves oxidative and non-oxidative processes and could be augmented by opsonization and T-cells’ cytokines [8]. Therefore, this study targeted to evaluate the role of IL-2, IL-4, IL-10, and IL-13 cytokine mRNA expression among the suspected cases of respiratory symptoms of *P. jirovecii* infection.

## Methods

### Design and setting

The present study was a cross-sectional analytic study undertaken in Aseer Central Hospital, Khamis Mushayt General Hospital, two tertiary care centers, and the College of Applied Medical Science, King Khalid University, Aseer region, southern Saudi Arabia, between January 2019 and August 2019.

### Study population

Based on the prevalence of the disease (30.3%) [18], the size of the study population was computed by using the formula, n = Zα^2^ (p x q)/D^2^. Initially the study cohort included 100-suspected PCP cases and further, we confirmed the positive cases by IFAT technique and 100 healthy controls. A written consent was obtained from all study participants. Essential clinical manifestations, radiological findings, microbiological and immunological findings from PCP cases attending Aseer Central Hospital from January 2019 to August 2019 were collected directly from patient charts.

PCP suspected cases who reported having regular respiratory symptoms like breath rapidity, pain in the chest, cough, and interstitial pulmonary infiltrates in X-ray or CT scan were included. The clinical outcome and clinical history of the suspected PCP cases were recorded.

Pneumocystis detection was done by reference diagnostic test methods such as immune fluorescent staining (IFAT) using the Merifluor DFA immunofluorescence test performed by following the manufacturer’s provided protocol (Meridian Bioscience, Inc., Cincinnati, Ohio). Other staining diagnostic techniques, like Gomorimethanamine silver staining (GMSS), Giemsa staining, Toluidine blue O (TBO), as well as Pneumocystis RT-PCR were used [19] to detect the Pneumocystis.

### Total RNA extraction and cDNA synthesis

3 ml of peripheral blood samples were withdrawn from suspected cases of PCP as well as from healthy control subjects. Total RNA extraction from the blood sample was executed using the QIAamp DNA Mini Kit (Qiagen, Germany) following instructions. The quality and quantity of total RNA were determined by the A260/280 ratio using the nano-spectrophotometer method.100 ng total RNA was taken to synthesize the cDNA by kit provided-method (Verso, Thermo Scientific, USA) for IL-2, IL-4, IL-10, IL-13 mRNA expression following the manufacturer’s protocol.

### Cytokine mRNA expression

Quantitative real-time PCR (qPCR) was performed to quantify the expression of Th1 and Th2 cell cytokines such as IL-2, IL-4, IL-10, IL-13 mRNA expression and glyceraldehyde-3-phosphate dehydrogenase (GAPDH) taken as a reference control to standardize the expression. The reaction was performed in a 25-μl reaction amount using the following program: 95 °C for 5 min, following 40 cycles of amplification, denaturation at 95 °C for 20 s, annealing (Table 1) at 58 °C - 60 °C for 30 s, and elongation at 72 °C for 30s using maxima SYBR green qPCR master mix technology. A melting curve was generated by programming fluorescent measurements every 1 °C from 35 °C until 95 °C to make sure a single PCR product.

**Table 1:** PCR primers sequences

Gene name	Primer sequence	Annealing temperature	Product length (bp)
IL - 2	FP: 5′-AACCTCAACTCCTGCCACAA-3′	60 °C	197
	RP: 5′-GCATCCTGGTGAGTTTGGGA-3′		
IL - 4	FP: 5′-CACAACTGAGAAGGAAACCTTCTG-3′	60 °C	253
	RP: 5′-CTCTCTCATGATCGTCTTTAGCCTTTC-3′		
IL - 10	FP: 5′-AGAACCT GAAGACCCTCAGGC-3’	60 °C	78
	RP: 5′-CCACGGCCTTGCTCTTGTT-3’		
IL - 13	FP: 5′-GCTCCTCAATCCTCTCCTGTT-3’	58 °C	485
	RP: 5′-GCAACTTCAATAGTCAGGTCC-3’		
GAPDH	FP: 5′-GAAGGTGAAGGTCGGAGTC-3’	60 °C	226
	RP: 5′-GAAGATGGTGATGGGATTTC-3’		

### Statistical analysis

The statistical analysis was executed using Graph Pad Prism version 6.05. The Mann – Whitney U test was used for nonparametric data, and t-test was performed for parametric data to compute the significant differences among the groups. QRT-PCR data was performed by the relative cycle threshold (Ct) method, and all samples were examined in triplicate. IL-2, IL-4, IL-10, and IL-13 mRNA expression levels were calculated by the relative quantification method using the 2^–(ΔΔCt)^ method [20]. Results more than or less than 1 were taken to indicate for up-regulation or down-regulation of mRNA expression. All the values were standardized compared to the normal control values, represented as a value of 1. *P*-value ≤0.05 was considered significant.

## Results

### Demographic and clinical features of study participants

**Fig. 1:**
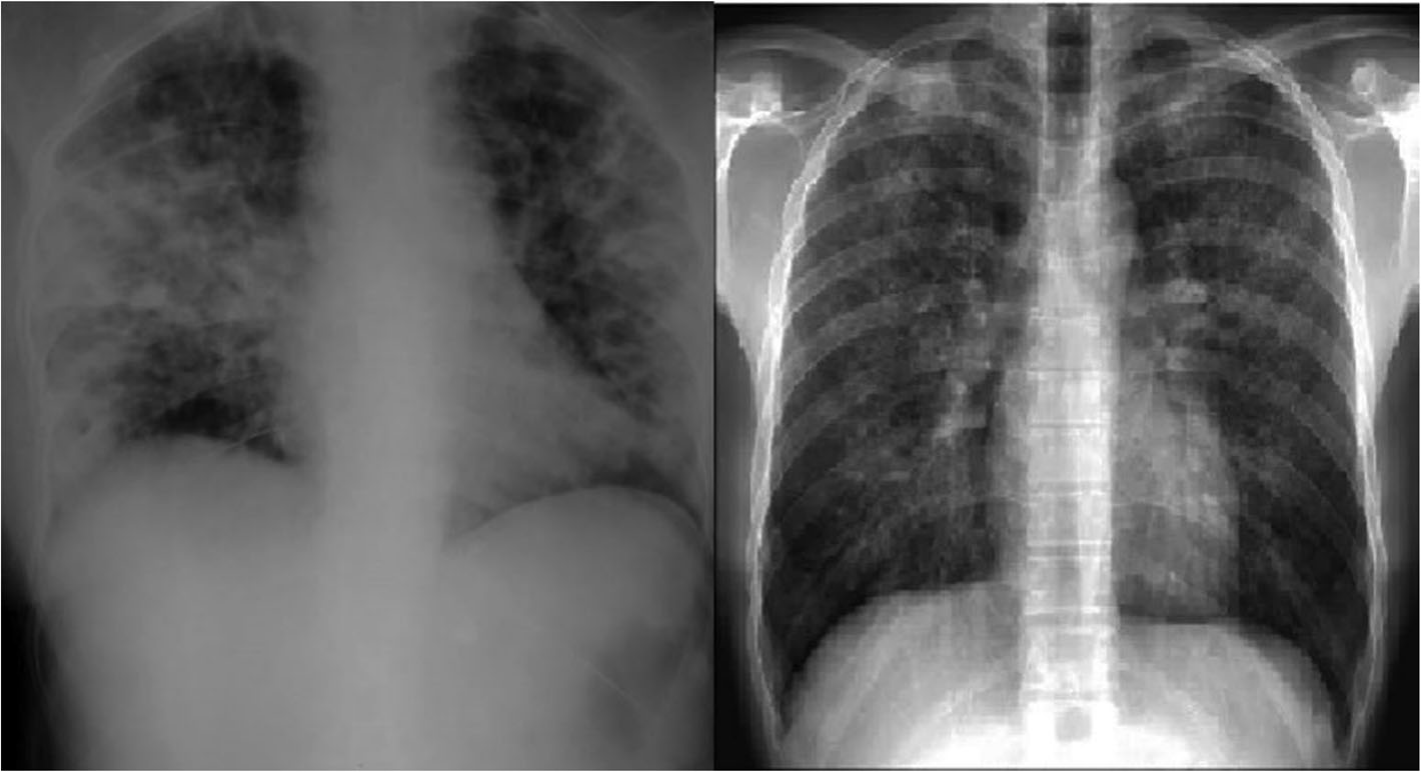
X-ray presentation of PCP positive patients showing multiple pulmonary involvement with massive pulmonary infiltrates

The present study included 200 study subjects. Among them, 100 were suspected cases of PCP, and 100 were healthy controls. Among the suspected cases of PCP, 59% were males and 41% were females, while among the healthy controls, 55% were males and 45% were females. Two age groups were determined based on the mean age of all study participants, < 50 years of age group cases were 45%, while > 50 years of age group cases were 55%, while in healthy controls < 50 years of age group healthy controls were 48% while > 50 years of age group of healthy controls were 52%. As shown in Table 2, all suspected cases had cough (100%), 92% had fever, 80% had weight loss, 76% had dyspnea, and 54% had night sweats, which was the most common clinical manifestation of PCP cases. 62 and 75% suspected cases of PCP had crept and wheezing, respectively. Chest X-ray showed multiple pulmonary findings, and the most common was pulmonary infiltrates (Fig. 1).

**Table 2:** Demographic and clinical characteristics of study subjects and the relative expressions of IL-2, IL-4, IL-10, and IL-13 mRNAs among Pneumocystis pneumonia (PCP) cases compared to healthy controls

Variables	No. of PCP cases	No. of healthy controls	IL-2 mRNA expression (Mean + SD)	*p*-value	IL-4 mRNA expression (Mean +SD)	*p*-value	IL-10 mRNA expression (Mean +SD)	*p*-value	IL-13 mRNA expression (Mean +SD)
Overall	100	100	5.72+ 3.16	–	4.35+ 4.20	–	3.77+ 2.99	–	7.59+ 4.82
Gender									
Males	59	55	5.70+ 3.27	0.57	3.91+ 3.62	0.39	3.37+ 2.68	0.20	8.0+ 4.80
Females	41	45	5.74+ 3.03		4.99+ 4.89		4.35+ 3.64		7.0+ 4.85
Age (in years)									
< 50 years	45	48	5.43+ 3.34	0.16	3.97+ 4.51	0.10	3.48+ 2.60	0.56	7.65+ 4.79
> 50 years	55	52	5.95+ 3.01		4.66+ 3.94		4.01+ 3.28		7.55+ 4.89
Cough									
Yes	100	NA^a^	5.72+ 3.16	–	4.35+ 4.20	–	3.77+ 2.99		7.59+ 4.82
No	0	NA	–		–		–		–
Fever									
Yes	92	NA	5.80+ 3.16	0.23	4.40+ 4.27	0.65	3.81+ 3.01	0.71	7.88+ 4.79
No	8	NA	4.71+ 3.17		3.79+ 3.47		3.36+ 1.60		4.34+ 4.10
Weight loss									
Yes	80	NA	5.82+ 3.16	0.38	4.77+ 4.12	0.002	3.99+ 2.96	0.02	7.74+ 4.93
No	20	NA	5.29+ 3.20		2.66+ 4.17		2.89+ 3.03		7.56+ 4.82
Dyspnea									
Yes	76	NA	5.77+ 3.25	0.92	4.94+ 4.47	0.003	4.15+ 3.21	0.01	7.57+ 4.82
No	24	NA	5.54+ 2.89		2.49+ 2.47		2.56+ 1.70		7.68+ 4.92
Night sweats									
Yes	54	NA	6.37+ 3.68	0.06	4.56+ 4.16	0.39	4.62+ 3.60	0.006	7.88+ 4.58
No	46	NA	4.95+ 2.20		4.10+ 4.27		2.77+ 1.58		7.26+ 5.11
Crept									
Yes	62	NA	6.35+ 3.32	0.007	5.0+ 4.86	0.01	4.16+ 3.29	0.09	8.01+ 5.08
No	38	NA	4.68+ 2.60		3.28+ 3.49		3.13+ 2.23		6.91+ 4.33
Wheezing									
Yes	75	NA	6.30+ 3.32	0.001	4.87+ 5.11	0.98	4.06+ 3.06	0.007	7.98+ 5.01
No	25	NA	3.96+ 1.70		4.17+ 3.87		2.92+ 2.63		6.45+ 4.08
Chest X-ray									
Central perihilar infiltrates	19	NA	7.59+ 2.83	< 0.0001	7.55+ 4.67	< 0.0001	6.11+ 4.05	< 0.0001	9.05+ 5.03
Patchy infiltrates	8	NA	9.88+ 5.12		3.46+ 1.98		5.40+ 3.63		7.30+ 4.30
Consolidation	3	NA	5.73+ 1.0		2.94+ 0.80		5.50+ 4.83		13.40+ 7.45
Hilar lymphadenopathy	3	NA	6.62+ 2.05		5.87+ 0.63		5.02+ 2.47		5.69+ 4.13
Pneumothorax	1	NA	5.38		1.08		1.67		5.09
Pleural effusion	1	NA	6.54		14.22		0.30		14.62
No	65	NA	4.61+ 2.37		3.41+ 3.84		2.83+ 1.88		6.95+ 4.57

^a^*NA* Not applicable

### Cytokine IL-2 mRNA expression

Increased mRNA expression of IL-2 (5.72 fold) was observed in suspected cases of PCP compared to healthy controls (Table 2). Significantly increased expression of IL-2 mRNA was associated with crept wheezing and chest X-ray findings. Patients who had a clinical feature like crept showed 6.30 fold IL-2 mRNA expression, while patients with no crept showed 4.68 fold IL-3 mRNA expression, and the differences among them were found to be significant (*p*=0.0007). Patients findings like wheezing showed 6.30 fold IL-3 mRNA expression, while patients without wheezing showed 3.96 fold IL-2 mRNA expression (*p*=0.001). Patient’s chest X-ray findings showed central perihilar infiltrate (7.59 fold), patchy infiltrate (9.98 fold), consolidation (5.73 fold), hilar lymphadenopathy (6.62 fold), pneumothorax (5.28 fold), pleural effusion (6.54 fold) showed higher expression of IL-2 mRNA expression compared to those patients did not show any such findings in X-ray (4.61 fold), and differences among them were found to be statistically significant (*p*< 0.0001).

### Cytokine IL-4 mRNA expression

IL-4 mRNA expression was 4.35 fold higher among the cases compared to healthy controls (Table 2). Higher expression of IL-4 mRNA was found to be significantly associated with weight loss (*p*=0.002), dyspnea (*p*=0.003), crept (*p*=0.01), and chest X-ray findings (p< 0.0001). Cases with weight loss, dyspnea, crept had 4.77 fold, 4.94 fold, 5.0 fold IL-4 mRNA expression, while cases with no weight loss, dyspnea, crept showed 2.66 fold, 2.49 fold, 3.28 fold IL-4 mRNA expression, respectively, which is lower compared to counterpart. Cases with different findings in X-rays such as central perihilar infiltrate, patchy infiltrate, consolidation, hilar lymphadenopathy, pneumothorax, pleural effusion showed 7.55 fold, 3.46 fold, 2.94 fold, 5.87 fold, 1.08 fold, 14.22 fold, respectively.

### Cytokine IL-10 mRNA expression

Overall, 3.77 fold increased IL-10 mRNA expression was observed among the cases compared to healthy controls (Table 2). Significantly higher expression of IL-10 mRNA was found to be associated with weight loss (*p*=0.02), dyspnea (*p*=0.01), night sweats (*p*=0.006), wheezing (*p*=0.007), and different findings of chest X-ray (*p*< 0.0001). Patients who had weight loss had 3.99 fold IL-10 mRNA expression, dyspnea had 4.15 fold IL-10 mRNA expression, night sweat had 4.62 fold IL-10 mRNA expression, and wheezing had 4.06 fold IL-10 mRNA which is higher compared to counterpart. Patient’s chest X-ray findings such as central perihilar infiltrate, patchy infiltrate, consolidation, hilar lymphadenopathy had higher IL-10 mRNA expression comparatively to other findings.

### Cytokine IL-13 mRNA expression

Increased mRNA expression of IL-13 (7.59 fold) was observed among the cases compared to healthy controls (Table 2). A significant association of IL-13 mRNA was observed in cases with fever (*p*=0.02). Cases with fever showed 7.88 fold higher IL-13 mRNA expression, while cases without fever showed 4.34 fold IL-13 mRNA expression, while no such association was observed with other clinical findings.

### PCP detection by IFAT and cytokine mRNA expression

**Fig. 2:**
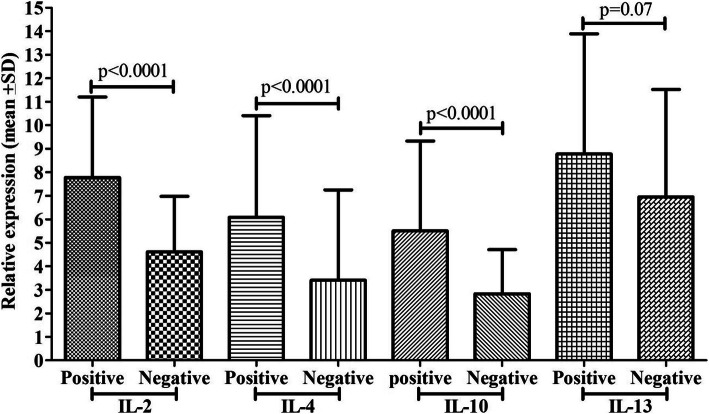
Comparison of cytokine mRNA expression (IL-2, IL-4, IL-10, and IL-13) between PCP positive and negatives cases by IFAT

Suspected cases of PCP were detected by the IFAT stain, and 35% were observed to be positive, and 65% were negative (Fig. 2). The 35% positive cases showed 7.78 fold IL-2 mRNA expression, 5.51 fold IL-10 mRNA expression, 6.08 fold mRNA expression, and 8.78 fold IL-13 mRNA expression. In comparison, 65% of negative cases showed 4.61 fold IL-2 mRNA expression, 2.83 fold IL-10 mRNA expression, 3.41 fold mRNA expression, and 6.95 fold IL-13 mRNA expression, respectively lower compared to its counterpart and differences among cytokines were found to be significant except IL-13 mRNA expression.

### PCP detection by GMSS and cytokine mRNA expression

**Fig. 3:**
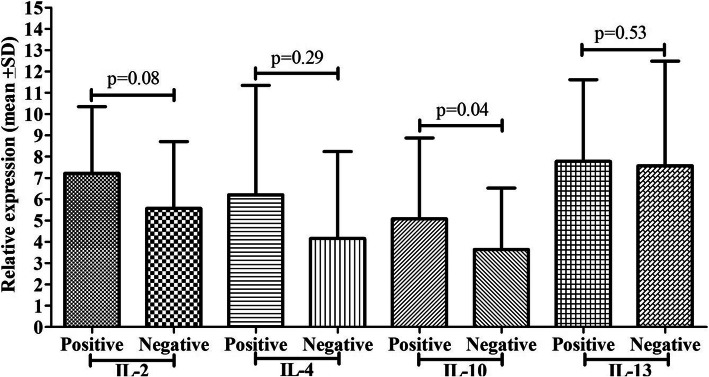
Comparison of cytokine mRNA expression (IL-2, IL-4, IL-10, and IL-13) between PCP positive and negatives cases by Giemsa

GMSS stain showed 15 (42.86%) positive cases out of 35 positive cases confirmed by IFAT for PCP, and others were negative (Fig. 3). Cytokines mRNA expression such as IL-2 (*p*=0.001) and IL-10 (*p*=0.004) showed a significant difference among the groups, while IL-4 and IL-13 did not show any significant difference. PCP positive cases by GMSS stain showed 8.37 fold IL-2 mRNA expression, 5.56 fold IL-10 mRNA expression, while negative cases showed 5.25 fold IL-2 mRNA expression, 3.45 fold IL-10 mRNA expression. Positive cases by GMSS had 5.23 fold IL-4 mRNA expression, 8.43 fold IL-13 mRNA expression, while negative cases for PCP by GMSS had 4.19 fold IL-4 mRNA expression and 7.45 fold IL-13 mRNA expression.

### PCP detection by Giemsa and cytokine mRNA expression

**Fig. 4:**
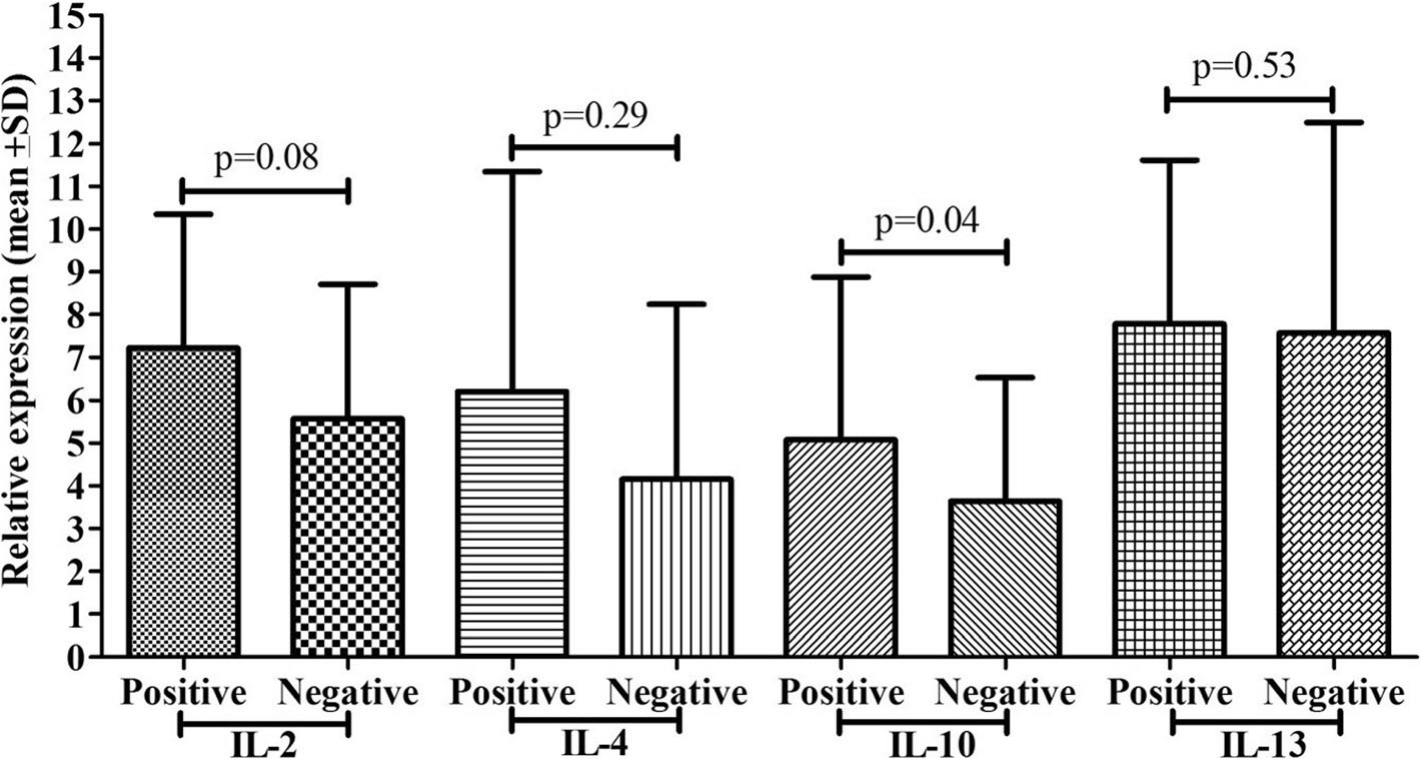
Comparison of cytokine mRNA expression (IL-2, IL-4, IL-10, and IL-13) between PCP positive and negatives cases by Giemsa

Giemsa stain showed 8 (17.6%) positive cases out of 35 positive cases confirmed by IFAT for PCP, and others were negative (Fig. 4). No such significant differences were reported in cytokine mRNA expression among the groups except IL-4 mRNA expression (*p*=0.04). Positive cases for PCP by Giemsa stain showed 6.21 fold IL-4 mRNA expression, while negative patients showed 4.16 fold IL-4 mRNA expression.

### PCP detection by TBO and cytokine mRNA expression

**Fig. 5:**
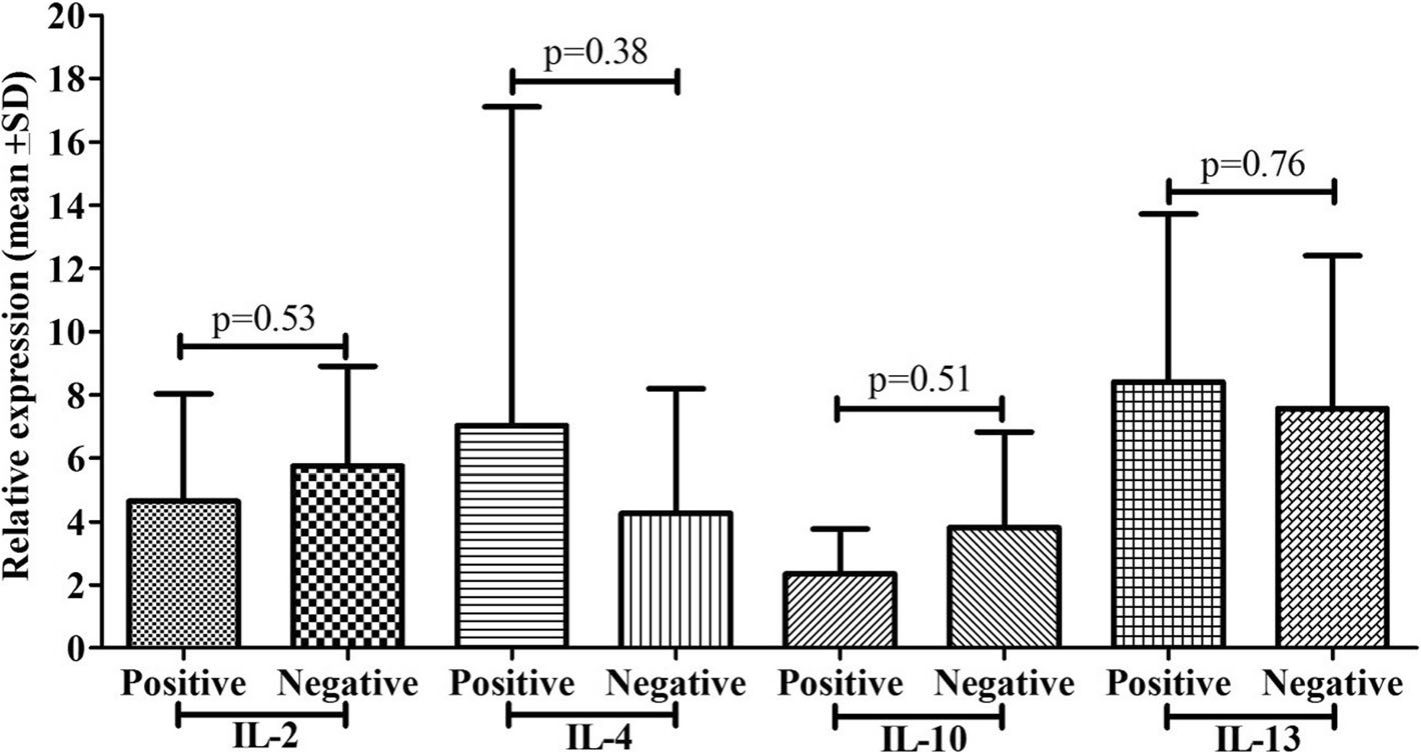
Comparison of cytokine mRNA expression (IL-2, IL-4, IL-10, and IL-13) between PCP positive and negatives cases by TBO

TBO stain showed 1 (2.8%) positive cases out of 35 positive cases confirmed by IFAT for PCP, and others were negative (Fig. 5). No significant differences were found in the expression of IL-2, IL-4, IL-10, and IL-13 mRNA expression between the positive and negative detected cases of PCP.

**Fig. 6:**
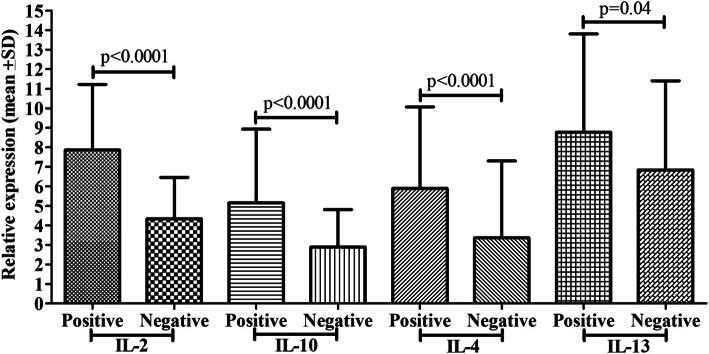
Comparison of cytokine mRNA expression (IL-2, IL-4, IL-10, and IL-13) between PCP positive and negatives cases by RT-PCR

### PCP detection by RT-PCR and cytokine mRNA expression

Detection of PCP was done by RT-PCR method, 39 (39%) of patients had PCP, and 59% suspected cases were negative for PCP (Fig. 6). PCP confirmed cases showed significantly higher Th1 (IL-2 and IL-10) and Th2 (IL-4 and IL-13) cytokine mRNA expression compared to negative cases. PCP positive cases showed 7.86 fold IL-2, 5.15 fold IL-10, 5.89 fold IL-4, 8.77 fold IL-13 mRNA expression, while negative cases showed 4.38 fold IL-2, 2.89 fold IL-10, 3.36 fold IL-4, 6.84 fold IL-13 mRNA expression and differences among them was found to be significant (*p*< 0.0001, *p*< 0.0001, *p*< 0.0001, *p*=0.04), respectively.

## Discussion

*P. jirovecii* is an opportunistic fungal pathogen confined to lung alveoli that causes pneumonia. Pneumocystis pneumonia remains a common cause of morbidity and death [21]. Activation of cellular effectors is necessary for the clearance of *P. jirovecii* by cytokine and the chemokine networks [3]. Iriart et al. stated that human *P. jirovecii* infection was connected with alterations in cytokine levels while comparing different groups of patients positive and negative for *P. jirovecii* infection [22]. The host immune response during Pneumocystis pneumonia comprises multifaceted interactions among T cells, alveolar macrophages (AM), neutrophils, and soluble arbiters that facilitate the pathogen’s approval as an elevated level of interleukin mRNA [23]. It has been validated that CD4+T lymphocytes have a crucial role in the infection pathogenesis and the opposite connection between the numerals of blood CD4+ lymphocytes and the risk of successive Pneumocystis pneumonia [24]. Cytokine and chemokine networks are essential of *P. jirovecii* approval by the commencement of these effectors. A study performed primarily in murine models showed an essential role for TNF-α or IL-10, two different cytokines in the T helper response [25].

An earlier study has done to check the mRNA expression level of cytokines of Th cells among the study cases for *P. jirovecii* infection, and further, the diagnosis was made by several methods such as IFAT, GMSS, Giemsa, TBO, and RT-PCR method to confirm the patients and to compare the diagnostic tests [19]. The present study observed more than 5 fold, 3 fold, 4 fold, 7 fold increased mRNA expression of IL-2, IL-10, IL-4, and IL-13, respectively, among the cases compared to healthy control subjects. Higher expression of IL-2, IL-10 mRNA was linked with cases that had crept, wheezing, and chest X-ray findings while lower expression in their counterparts comparatively. Besides, the higher expression of IL-10 mRNA was also significantly associated with the cases who had weight loss and dyspnea compared to its counterpart. Higher expression of IL-4 mRNA was associated with cases that had weight loss, dyspnea, crept, and chest X-ray findings, while higher expression of IL-13 mRNA was associated with cases who reported fever.

In adult patients, *P. jirovecii* colonization makes them cause infection and has been depicted with chronic obstructive airway disease [26]. In our study, clinical observations such as cases with cough (100%), cases with fever (92%), cases with unintentional weight loss (80%), and cases with dyspnea (76%) were connected with the risk of PCP and similar findings were also reported [27].. PCP positive patients had 85.7% typical interstitial pulmonary infiltrates consistently observed in chest X-ray findings. A similar study reported that pulmonary infiltrates were observed in 89.5% of patients, and 84.2% showed bilateral pulmonary infiltrates [28]. Chronic inflammatory disorder was explained by airflow limitation, which linked to a multifaceted inflammatory response in the lungs. Colonization of *P. jirovecii* infection in the lung tissue can cause pulmonary tissue damage and the deterioration of lung function through inflammation and the related inflammatory arbitrator. Therefore, *P. jirovecii* colonization influences disease progression [29].

PCR methods for the diagnosis of PCP in bronchoalveolar lavage fluid have shown good sensitivity and specificity. These authors have encouraged the use of clinical practice, radiography as well as laboratory findings of suspected patients for a definitive diagnosis of PCP [30]. Based on different diagnostic methods used to detect the *P. jirovecii* infection such as IFTA, GMSS, Giemsa, TBO, and RT-PCR methods. Two groups were made based on the positive and negative detection of *P. jirovecii* in suspected cases. Cytokine mRNA expression was evaluated and compared for the groups confirmed positive and negative for *P. jirovecii*. Cases that were positive for *P. jirovecii* by the IFTA method showed significantly higher IL-2, IL-10, and IL-4 mRNA expression levels than negative cases. Elevated expression of IL-2, IL-10 mRNA was detected in cases positive for *P. jirovecii* by GMSS method, while negative showed comparatively low mRNA expression. Positive cases for *P. jirovecii* by the Giemsa method only showed higher mRNA expression of IL-14 than its cases. In contrast, cases detected positive for *P. jirovecii* and negative for PCP by TBO method did not show any differences in cytokine mRNA expression. Cases reported positive for *P. jirovecii* by RT-PCR method showed significantly higher IL-2, IL-10, IL-4, and IL-13 mRNA expression compared to negative cases of PCP.

Write and colleagues revealed that T-cells play a decisive role in inflammation stimulation in feedback to Pneumocystis infection, inducing elevated levels of cytokine mRNA at sites of infection and acute alveolar inflammation [23]. Fungal or microbial infections connected with granulomatous inflammatory and depend on coordinated communication of innate and adaptive immune reactions by Th1 response [31]. This defensive immune reaction involves the secretion of cytokines, which leads the classical commencement of macrophages and their recruitment at the site of infection [31, 32]. Higher IL-4, IL-10, and IL-13 levels were observed to be associated with inadequate IFN-γ production, and otherwise, activation of macrophages dominant to uncontrolled fungal infection [33, 34] as well as Cenci et al. in 1998 reported that mice infected with fungal pathogens are susceptible to produce an IL-10 and IL-4 [35]. Th2-mediated allergic responses to fungal in mice, resulting in repeated lung exposure to fungal strains, produce IL-4 and IL-13 (Lilly LM et al., 2012) as well as the intratracheal direction of fungal spores induced allergic asthma in mice [36]. Improved understanding of cytokine expression may be a clue of patients’ treatment having infectious diseases.

### Limitations

Control individuals in the present study have been clearly chosen on the absence of symptoms of PCP. Healthy controls who have been included were those who did not show any respiratory symptoms in the last 6 months and any other complications related to the chest or any medication received in the last 6 months. Therefore, it is one of the limitations of the present study that colonization, infection or PCP disease should be discriminated from each other. However, our current methodology cannot reach to this level of discrimination, given the fact that it was not part of the original aims. Definitive diagnosis of PCP requires the demonstration of Pneumocystis in the lungs of a patient with compatible pulmonary signs and symptoms, nevertheless, pneumocystis has remained tenacious to study [37].

Another limitation of this study as for similar studies, is that different diagnostic methods revealed different results [38]. This is obvious and acceptable given the fact that these methods have different sensitivities and specificities and work on a specific part of the fungus or the host immune response [37, 39]. For instance, the Calcofluor white and GMS stains have better results for routine use in a clinical laboratory [38]. Pneumocystis species have a life cycle with both mitotic and meiotic phases analogous to other ascomycetous fungi, but with unique adaptations to interact with host cells and proliferate within the unique niche of the mammalian lung.

## Conclusions

Identifying specific markers and molecular signatures is an important step that will support clinical diagnosis to improve patient’s management. *P. jirovecii* patients presented high IL-2, IL-4, IL-10, and IL-13 mRNA expression compared to negative controls. Increased expression of cytokines may be indicative of infection severity and could help in patients’ management. The study observed increased IL-2, IL-4, IL-10, and IL-13 mRNA expression in suspected cases of *P. jirovecii* infection. Confirmed cases of *P. jirovecii* showed higher IL-2, IL-4, IL-10, and IL-13 mRNA expression comparatively to negative cases. The present study did not explore TNF alpha and IFN-ϒ since these markers have been studied [40–42]. Our study focused on exploring IL-2, IL-4, IL-10, and IL-13 mRNA expression levels which have not been investigated in PCP extensively and they fulfil the diagnostic aims among the studied PCP population in comparison to healthy controls. Increased expression of cytokines may be indicative of infection severity (hypoxia) and could help in patients’ management. Monitoring the expression of interleukins may help plan a treatment strategy and overcome the problem of treatment failure and therapy response. Cytokines could open the avenues for fungal infections’ treatment of fungal infections.

## Data Availability

The datasets used and/or analyzed during the current study are available from the corresponding author on reasonable request.

## Abbreviations

PCP: Pneumocystis pneumonia
PJP: *Pneumocystis jirovecii*
IL-: Interleukins
mRNA: Messenger RNA
IFAT: Immune-fluorescent staining
GMSS: Gomorimethanamine silver staining
